# Trauma Outcomes in Pediatric Nonfatal Road Traffic Accidents

**DOI:** 10.3390/children11040425

**Published:** 2024-04-03

**Authors:** Ștefan Popa, Diana Bulgaru-Iliescu, Carmen Iulia Ciongradi, Adrian Onisim Surd, Iuliana-Laura Candussi, Irene Paula Popa, Ioan Sârbu

**Affiliations:** 1 2nd Department of Surgery–Pediatric Surgery and Orthopedics, “Grigore T. Popa” University of Medicine and Pharmacy, 700115 Iași, Romania; stefan.popa@umfiasi.ro (Ș.P.); sarbu.ioan@umfiasi.ro (I.S.); 2 3rd Department of Medical Specialities–Legal Medicine, “Grigore T. Popa” University of Medicine and Pharmacy, 700115 Iași, Romania; diana.bulgaru@umfiasi.ro; 3 Department of Pediatric Surgery, “Iuliu Hațieganu” University of Medicine and Pharmacy, 400347 Cluj-Napoca, Romania; adisurd@elearn.umfcluj.ro; 4 Clinical Surgery Department, Faculty of Medicine and Pharmacy, “Dunărea de Jos“ University, 800008 Galați, Romania; iuliana.candussi@ugal.ro; 5 Department of Physiology, “Grigore T. Popa” University of Medicine and Pharmacy, 700115 Iași, Romania

**Keywords:** road traffic accidents, children, adolescents, orthopedic interventions, fractures, safety

## Abstract

Background: By 2025, road traffic injuries are projected to rank third in the global burden of disease, posing a significant challenge that affects health, social well-being, and economic aspects. According to data from the Romanian Police National Statistics Center, there have been an average of 342 traffic accidents per year involving pediatric patients over the past 10 years. Materials and Methods: A retrospective research study was conducted, encompassing 358 cases of road traffic accidents identified for the study, with data collected over a span of eight years, and with the aim of analyzing the types of injury and treatment methods in relation to age and sex, while also focusing on the duration of hospitalization and the occurrence of complications. Results: An oscillating trend is observed from 2015 to 2020, with its lowest value recorded in 2017 at around 6.8% and its peak in 2019 at 20.1%. Notably, post-pandemic (COVID-19), the cases underwent a substantial decline of approximately 60%. At least 78.7% of those who did not undergo orthopedic reduction required surgery, whereas among those who underwent orthopedic reduction, only 23.4% needed surgery. Regarding the frequency of complications 17.3% of the total cases experienced complications. Conclusions: According to our findings, age has a significant effect on the type of accident (*p* < 0.05). Complications occurred in 17.3% of patients, most commonly surgical (24 cases, 38.7%), orthopedic (17 cases, 27.4%), and neurological (15 cases, 24.2%).

## 1. Introduction

Road traffic accidents (RTAs) are regarded as integral components of “development diseases” often stemming from the rise in motor vehicle numbers, population densities, and environmental alterations [1]. Despite their significance, RTAs constitute a major yet frequently overlooked public health issue, entailing elevated rates of both mortality and morbidity on a global scale [2]. They constitute a pervasive global challenge, impacting health, social well-being, and economic spheres, resulting in an annual toll of up to 50 million injuries [3]. By 2025, road traffic injuries are expected to take third place in the rank order of disease burden [4].

**Figure 1:**
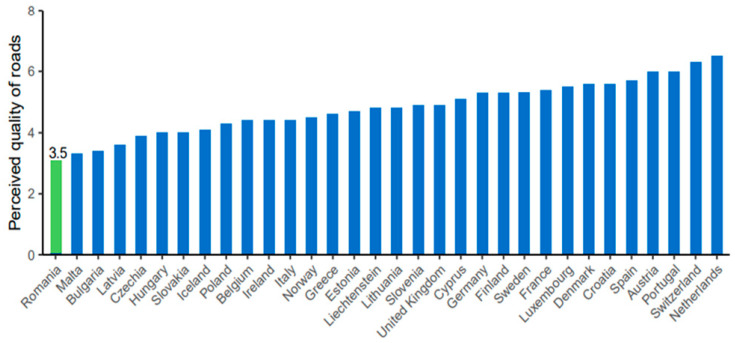
Perceived quality of the road infrastructure (1 = extremely poor, 7 = among the best in the world). Source: World Economic Forum, Executive Opinion Survey (2019).

One of the leading causes, as indicated, is the average perceived quality of road infrastructure in the EU, which is above 4 on a scale of 1 to 7 (where 1 represents extremely poor and 7 represents among the best in the world). In Romania, it is around 3.5, the lowest in the entire Europe [5] (Figure 1).

The term ‘accident’, as defined in 1956, implies an unavoidable and unpredictable situation, a happening that cannot be averted. In contrast, the 2004 WHO Report on Road Traffic Injury Prevention favors the use of the term “crash” [6].

In the United States, the overall pediatric trauma survival rate varies between 80% and 95% [7,8,9,10,11], and statistics from the Netherlands indicate that for every child killed in a road traffic accident (RTA), another 42 are seriously injured [12]. Based on the information from the Romanian Police National Statistics Center, there has been an average of 342 traffic accidents involving pediatric patients over the past 10 years [13].

Nonfatal injuries, even those considered minor, can result in significant short and long-term physical and psychological outcomes that are linked to substantial losses for individuals in all aspects of life, including their quality of life. Such injuries constitute a major cause of temporary or permanent disability and exert a notable negative impact on families and community networks [7,8,9,10,14,15].

Children, especially, are a vulnerable group in the context of road traffic. Younger children, in particular, face an increased risk of being run over by a vehicle. Despite having the necessary motor skills to walk on streets, they lack cognitive, sensory, and behavioral perception skills to comprehend traffic and associated risks [16,17]. Being run over by a vehicle is the leading cause of death and disability for children in numerous countries [18], and the resulting injuries often tend to be more severe than those suffered when inside motor vehicles [19].

Furthermore, children experience different types of injuries compared to adults. This is attributed to their lower mass, resulting in reduced kinetic force and lower-intensity accidents. Additionally, children often sit in the rear seats of vehicles, providing them with more protection. As a result, injury patterns differ between children and adults. Children tend to have fewer thoracic, intra-abdominal, pelvic, and long bone injuries, and they typically exhibit a lower Injury Severity Score (ISS) despite a higher Glasgow Coma Scale score [17,20]. These results may be explained by a better adaptive process and greater physiological plasticity, leading to a more effective response to trauma in children. This adaptability allows children to recover more successfully without permanent sequelae [21]. However, in cases where sequelae do occur, conducting a Personal Injury Assessment (PIA) for children becomes challenging. It involves clarifying concerns related to permanent disabilities and future needs, which are difficult to predict for the child’s lifetime.

The aim of this study was to analyze the relationship between age and sex and the types of injuries and treatment methods, while also focusing on the duration of hospitalization and the occurrence of complications. We additionally grouped the patients into four specific age categories, considering that the majority of existing literature has primarily focused on age-specific grouping patterns [22,23].

## 2. Materials and Methods

In this study, we took a close look back at the years from 2015 to 2022, gathering information from the Emergency Department (ED) at “St. Mary’s” Emergency Clinical Hospital for Children and the Pediatric Orthopedics Department. Approval for the study protocol was obtained from the administration of the “St. Mary’s” Clinical Emergency Hospital for Children under the designated approval number 23,177 dated 10 July 2020.

“St. Mary’s” ED is the go-to hospital for children dealing with injuries in Iaşi County and its surroundings, covering both city and country areas. Information from the hospital’s computerized medical records was extracted to identify patients admitted to the ED for TAs. Our emphasis was primarily on patients with cause codes V00–V99. We even did a digital scan using keywords like “road traffic accidents” and “traffic accidents”. This verification was carried out by meticulously scrutinizing their medical data.

The hospital’s ED functions as the main public facility for referring pediatric injuries, offering highly specialized medical treatment for the city and territory of Iaşi, as well as its surroundings, encompassing both rural and urban regions. Information from the hospital’s computerized medical records was extracted to identify patients admitted to the ED for TAs. Our emphasis was primarily on patients with cause codes V00–V99. Furthermore, an electronic search by the keywords “road traffic accidents” and “traffic accidents” was conducted to uncover any potential occurrences of traffic accidents that may have been missed during the classification procedure, which relied on provided cause codes. This verification was carried out by meticulously scrutinizing their medical data.

A total of 358 cases of RTAs were identified for the study. The inclusion criteria for this study encompassed patients who experienced trauma due to road accidents and required hospitalization for their injuries. Conversely, patients with trauma thought to be less severe, which could be managed at home without hospitalization, were excluded from the study. Data collection involved obtaining information on demographics, detailed data on the types of injuries sustained, and the treatment interventions provided. The anatomical region affected was correlated with the different accident types, and the characteristics of the harm inflicted determined the classification of injury types.

To conduct the statistical analyses, participants were categorized into four age groups spanned from 0 to 4 years, 5 to 9 years, 10 to 14 years, and 15 to 17 years.

All the data from the study were analyzed using IBM SPSS Statistics 25 and illustrated using Microsoft Office Excel/Word 2013.

Quantitative variables were tested for normal distribution using the Shapiro–Wilk test and were written as averages with standard deviations or medians with interquartile ranges. Quantitative independent variables with a non-parametric distribution were tested between groups using Mann–Whitney U/Kruskal–Wallis H tests. Qualitative variables were written as absolute or relative frequencies and were tested between groups using Fisher’s exact tests.

Z-tests with Bonferroni correction were used to further detail the results obtained in the contingency tables. Throughout all analyses, a *p*-value of <0.05 was considered statistically significant.

A multinomial logistic regression was used to predict the type of accidents (considered as a dependent variable) using the age classes (considered as an independent variable). The reference category in the type of accidents was considered the pedestrian category, while the reference category in the age classes was the 15–17 year group. The performance of the prediction will be illustrated as odds ratios with 95% confidence intervals along with the significance value.

## 3. Results

**Figure 2:**
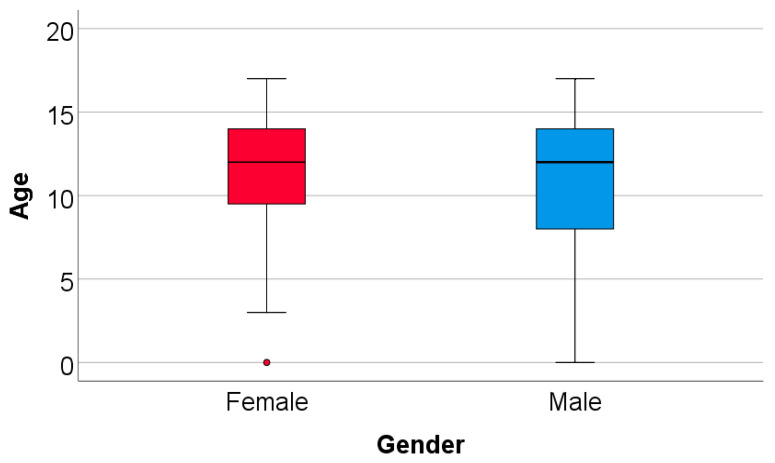
Box-plot representation of the patient’s age comparison according to gender.

The gathered data indicate a total of 358 RTAs occurring across a range of 8 years, with the youngest case being 1 month old and the oldest being 17 years old (Figure 2).

The mean age of the patients was 11.13 ± 4.37 years (median = 12 years), with most of the patients between 10 and 14 years (45.5%) or 15 and 17 years (23.7%). Most of the patients were male (77.7%) (Table 1).

**Figure 3:**
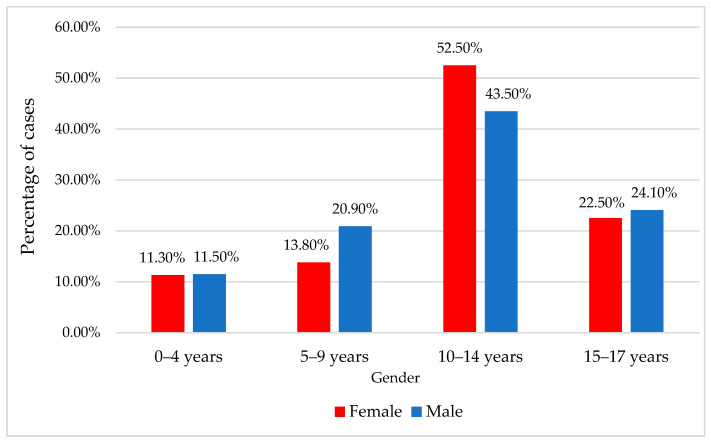
Distribution of the patient’s age categories according to gender.

The data from Table 1 and Figure 2 and Figure 3 illustrate the comparison and distribution of patient ages according to gender. The findings reveal that there was no significant difference in patient age between genders, whether analyzed as a continuous variable or as a categorical variable (*p* > 0.05). The majority of patients were in the range of 10–14 years, with female patients accounting for 52.5% and male patients for 43.5%. The median age for both genders was 12 years (Table 1, Figure 2 and Figure 3).

There were 8 children with an age below 1 year (the data show that one girl and 7 boys had an age below 1 year at the moment when the data were collected). And according to the box-plot figure, since the distribution of age in the boys’ group is extended towards the minimum age (age distribution in boys: median = 12 years, IQR = 8–14 years, lower limit for outliers: 25th percentile—1.5*IQR = 8–9 = −1), and the 7 boys aged below 1 year were not considered outliers in the box-plot figure, the <1 year age was not considered important (Figure 2). Of course, there was one case in the female patients detected as an outlier, only because of the lower IQR and higher 25th percentile (age distribution in girls: median = 12, IQR = 9.5–14 years, lower limit for outliers: 25th percentile—1.5*IQR = 9.5–6.75 = 2.75 years).

In the dataset, 271 patients were identified (constituting 76.1%) predominantly from rural areas, whereas only 23.9% originated from urban environments.

**Figure 4:**
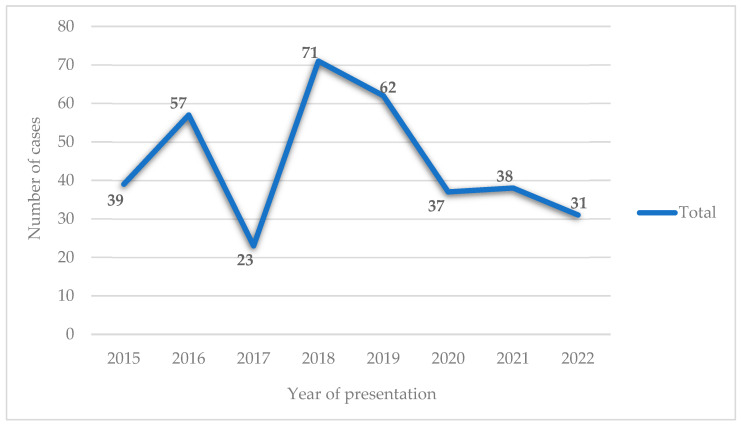
The occurrence rate of cases presented to the service in relation to the study years.

With a focus on the period from 2015 to 2022, we observed an oscillating trend between 2015 and 2020, reaching its lowest value in 2017 at around 6.8% (24 out of 358 cases) and its maximum value in 2019, reaching 20.1% (71 out of 358 cases). It is also noted that post-pandemic (COVID-19), the cases experienced a significant drop to approximately 50% of the previous years and maintained a downward trend until 2022 (Figure 4).

Throughout the study period, seasonal variation marked a peak frequency of cases in June, accounting for 15.3% (55 out of 358 cases), corresponding to the beginning of summer vacation. Following this, September showed a rate of 10% (26 out of 358 cases), coinciding with the start of the school year. In contrast, the lowest number of cases was recorded during the winter month of January, with a rate of 1.7% (6 out of 358 cases).

Regarding pathological personal histories, it was found that 85.4% of patients (equivalent to 306 out of 358) had no prior pathologies before their presentation. The remaining 14.6% (equivalent to 52 out of 358) exhibited diverse antecedents, including allergies and conditions related to the heart, gastrointestinal, nutrition, neurology, as well as orthopedic issues. There were 3 cases with 3 different pathologies: 1 case with respiratory history, 1 case with gastroenterological history, and 1 case with diabetes mellitus, grouped together as others. As the data shows, the frequencies of personal pathological history or the type of history were not significantly different between age groups in female or male patients (*p* > 0.05, Fisher’s exact tests) (Table 2 and Table 3).

Differences in hospitalization period between age categories were significant according to the Kruskal–Wallis H test (*p* = 0.043), and post-hoc Dunn–Bonferroni tests show that only patients aged between 15 and 17 years had a significantly higher hospitalization period (median = 8 years, IQR = 5–11 years) than patients aged between 0 and 4 years (median = 6 years, IQR = 3–8 years) (*p* = 0.034).

**Figure 5:**
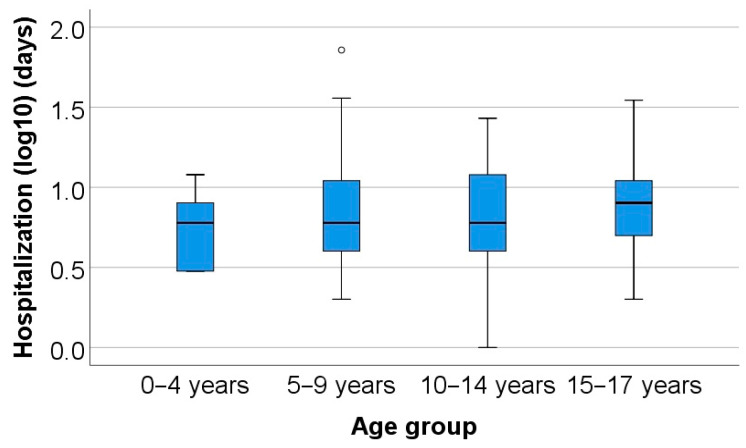
Comparison of hospitalization period according to age categories (period represented using the base10 logarithm).

The outliers for each age group, as depicted in the box plot representation, are as follows: In the 5–9-year age group, there was 1 case with 72 days of hospitalization and 2 cases with 36 days each. In the 10–14-year age group, there were 2 cases with 27 days of hospitalization. For the 15–17-year age group, there were 2 cases with 35 days and 2 cases with 29 days of hospitalization (Figure 5).

**Figure 6:**
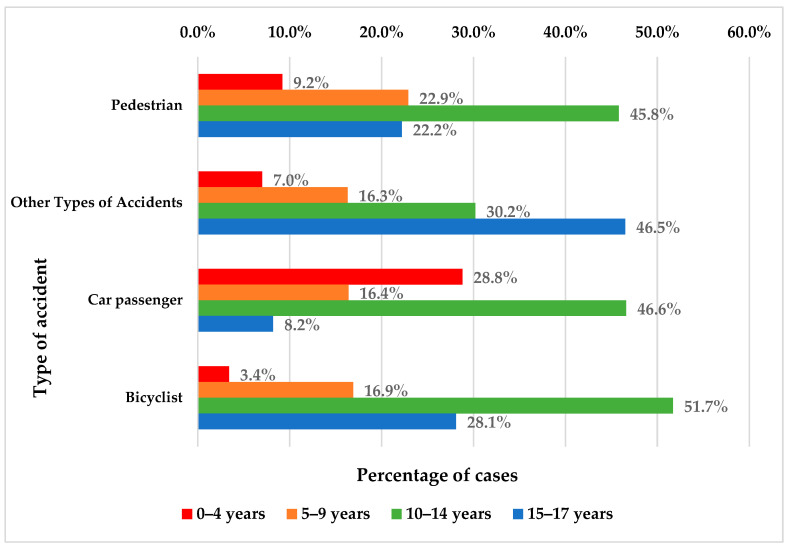
Distribution of the patients according to age and type of accident.

Patients aged between 0 and 4 years were more frequently car passengers than pedestrians/involved in other types of accidents/bicyclists (28.8% vs. 9.2%/7%/3.4%), while patients aged between 15 and 17 years were more frequently involved in other types of accidents or bicyclists than pedestrians or car passengers (46.5%/28.1% vs. 22.2%/8.2%) (*p* < 0.001) (Figure 6).

Age has a significant effect on the type of accident (*p* < 0.001). Specifically, age significantly influences the likelihood of patients experiencing other types of accidents and being car passengers (decrease). Not all types of accidents are significantly influenced by age, such as being a cyclist or a passenger in a car, where the relationship is not statistically significant.

For cyclists, the coefficient for age is 0.050 with a *p*-value of 0.161, suggesting that although there is a trend for the likelihood of being a cyclist to increase with age, this effect is not statistically significant.

For car passengers, the coefficient is −0.129, significant at the 0.000 level, indicating that the likelihood of being in this category decreases with age. An Exp(B) of 0.879 indicates a decrease of 12.1% in the chances for each additional year compared to being a pedestrian.

For other types of accidents, the coefficient for age is 0.532, significant at the 0.000 level, indicating that the likelihood of experiencing this type of accident significantly increases with age. An Exp(B) of 1.703 suggests that for each additional year, the chances of having this type of accident increase by 70.3% compared to being a pedestrian.

Based on a multinomial logistic regression model presented in Table 4, the age classes significantly predict the accident types. The data shows the following: children aged 15–17 years have significantly higher odds of having other types of accidents than being pedestrians in comparison to children aged 10–14 years (*p* = 0.005, OR = 3.164 times (95% C.I.: 1.41–7.092)) or children with an age of 5–9 years (*p* = 0.031, OR = 2.941 times (95% C.I.: 1.102–7.874); children with an age of 0–4 years (*p* < 0.001, OR = 8.5 times (95% C.I.: 2.829–25.542)) or with an age of 10–14 years (*p* = 0.039, OR = 2.752 times (95% C.I.: 1.054—7.186) have significantly higher odds of having an accident as car passengers than being pedestrians in comparison to children with an age of 15–17 years (Table 4).

Using Fisher’s exact test, it was found that there is a significant correlation between the age of the participants and the types of accidents they are exposed to, suggesting that certain types of accidents are more likely to occur at certain ages (*p* < 0.001) (Table 5). However, no significant correlation was found between the participants’ gender and the type of accident, indicating that the distribution of accident types is relatively similar between men and women in this dataset (*p* = 0.090) (Table 6).

Another aspect is that patients aged between 0 and 4 years had more frequent lesions determined by an impact with an object in the car (because we found out that the majority of the car passengers do not have seatbelts or are not provided with special car seats being held by their parents (n = 50, 69.5%) than by an impact + falling or just falling (28.8% vs. 5.9%/1.5%), while patients aged between 15 and 17 years had more frequent lesions determined by falling or an impact with an object from the car (35.8% vs. 8.2%) (*p* < 0.001). Another significant finding was that 69.6% (62 out of 89) of cyclists involved in accidents were not wearing helmets at the time of impact.

**Figure 7:**
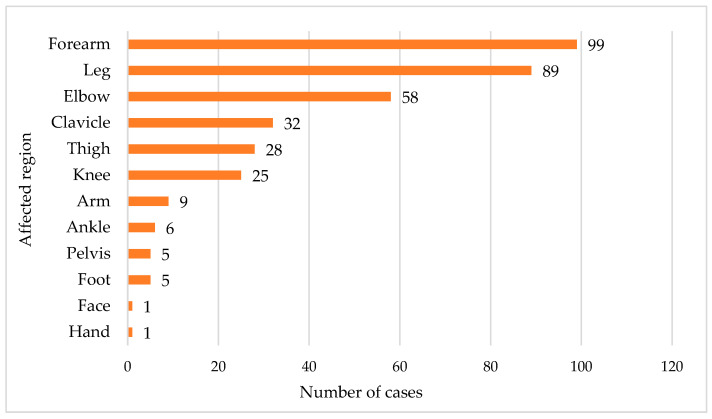
Distribution across affected segments in the cases of patients involved in the study.

Data from the observation sheets showed that the most impacted segment was the forearm, accounting for 99 out of 358 cases (28%). Conversely, the face and hand were the least affected, collectively making up less than 1% of the cases. The remaining segments ranged from 1.4% to 24.9%, as seen in Figure 7.

**Figure 8:**
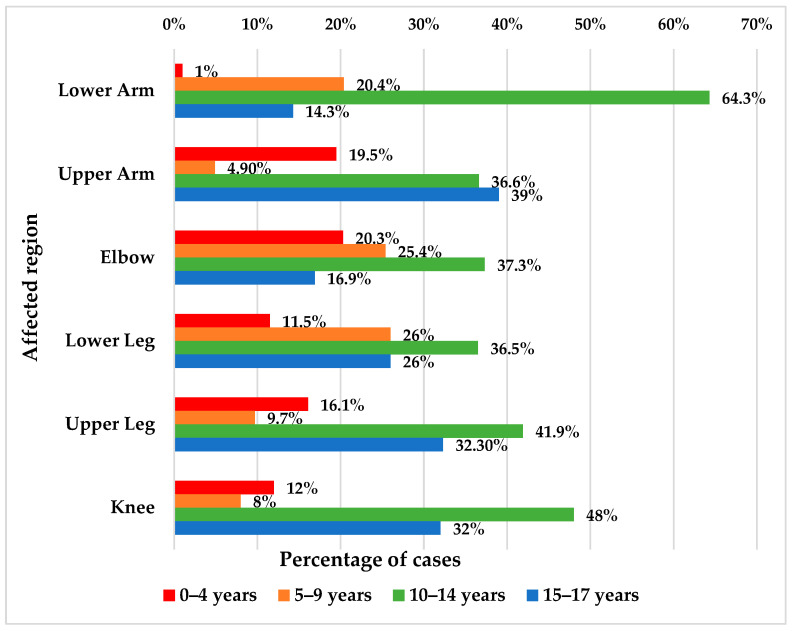
Distribution of the patients according to age and affected region.

Patients aged between 0 and 4 years were significantly more associated with lesions at the upper arm/elbow/lower leg/upper leg than at the lower arm (19.5%/20.3%/11.5%/16.1% vs. 1%), patients aged between 10 and 14 years were significantly more associated with lesions at the lower arm than at the upper arm/elbow/lower leg/upper leg (64.3% vs. 36.6%/37.3%/36.5%), and patients aged between 15 and 17 years were significantly more associated with lesions at the upper arm than at the lower arm (39% vs. 14.3%) (*p* < 0.001) (Figure 8).

**Figure 9:**
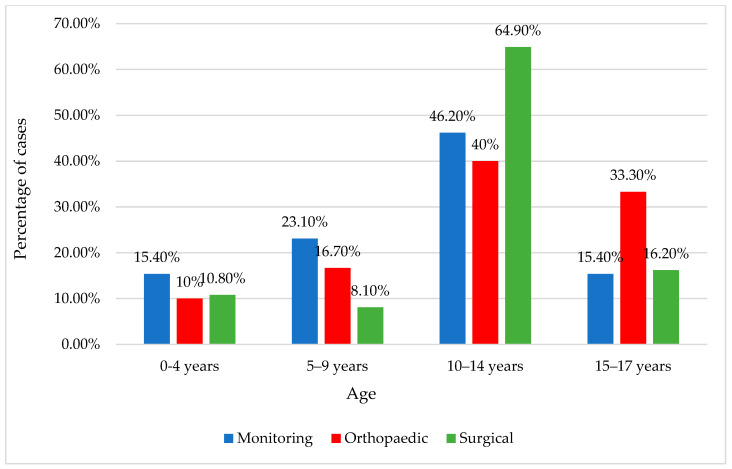
Distribution of the female patients according to age classes and treatment approach.

**Figure 10:**
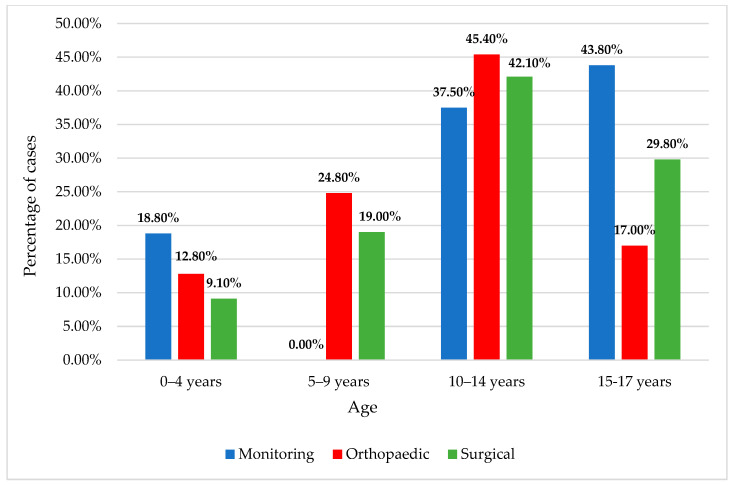
Distribution of the male patients according to age classes and treatment approach.

The majority of patients underwent orthopedic treatment (47.8%) or surgical intervention (44.1%), with an average hospitalization duration of 8.5 ± 6.6 days (median = 7 days) (Figure 9 and Figure 10). For those who did not undergo surgery, there remained a need for orthopedic reduction and immobilization, accounting for a substantial proportion, precisely 61.6%.

At least 78.7% of those who did not undergo orthopedic reduction required surgery, whereas among those who underwent orthopedic reduction, only 23.4% needed surgery. With *p* < 0.05, it is acknowledged that there is a significant association between orthopedic reduction and the necessity for surgery (χ^2^ = 103.54; df = 1; *p* < 0.001).

The majority of patients had associated injuries (265 cases, 74%), with the most frequent being excoriated wounds (104 cases, 29.1%), chest contusions (73 cases, 20.4%), abdominal contusions (71 cases, 19.8%), traumatic brain injuries (52 cases, 14.5%), other fractures (59 cases, 16.5%), limb contusions (34 cases, 9.5%), and hematological changes (30 cases, 8.4%).

Complications occurred in 17.3% of patients, most commonly surgical (24 cases, 38.7%), orthopedic (17 cases, 27.4%), and neurological (15 cases, 24.2%).

**Figure 11:**
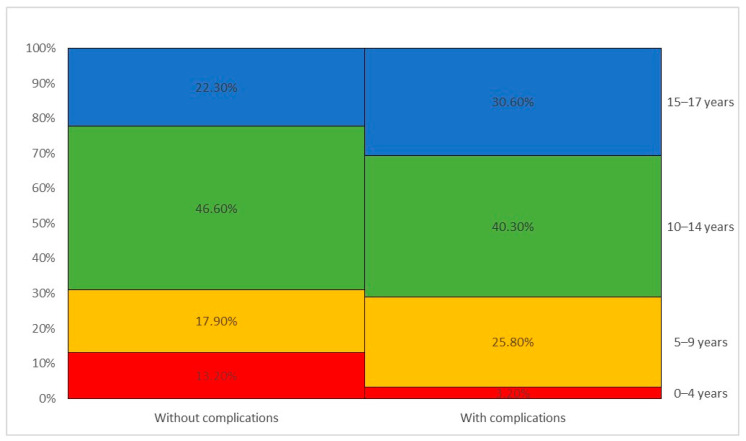
Complications among patients by age group.

Patients aged 0–4 years had significantly fewer complications compared to older age groups (3.2% vs. 13.2%) (*p* = 0.034) (Figure 11), while different types of accidents were not significantly associated with complication occurrence (*p* = 0.983 > 0.05).

Of those experiencing complications, a total of 32.2% suffered from postoperative wound dehiscence (N = 10) and wound infections (N = 10), while approximately a total of 9.7% exhibited osteoarticular injuries, such as posttraumatic shortening (N = 4) and genu varum (N = 2) (Table 7).

## 4. Discussion

In alignment with the demographic assessment conducted at the national level in Romania, during the year 2020, over half of the total newborns, specifically 51.6%, were male, indicating a sex ratio of 107 boys per 100 girls [23].

While a national-level gender difference may not be pronounced, a substantial shift is observed in the context of traffic accidents. Variations were noted in the distribution of sexes, with a male predominance in RTAs aligning with findings from several studies [24,25,26,27,28,29,30,31,32]. However, some studies did not observe such differences [12,13], while others reported a female predominance [33,34].

In our study, we did not detect any disparity between genders; however, certain studies indicate that women, particularly those over 14 years old, may sustain more severe injuries compared to men [28,33]. Conversely, other research has suggested that men tend to suffer more severe injuries [32]. Additionally, studies have highlighted differences in how men and women experience accidents.

It is noteworthy that a notable portion of accidents, approximately 31%, occurred in children under the age of 14. However, a clear shift in the frequency and nature of accidents was observed after the age of 15. Seasonal variation was evident, with a peak frequency of cases occurring in June, representing 15.3% (55 out of 358 cases). This increase may be linked to the favorable weather conditions typical of Eastern Europe, characterized by a temperate-continental climate that fosters pedestrian and cycling activities [35,36].

It is essential to emphasize that previous academic investigations have primarily focused on the fatal aspects of traffic accidents or solely on automobile collisions, thus distinguishing our study’s design in terms of its coverage and methodology.

Significant differences exist in long bone fractures, which account for 10%–25% of all pediatric traumatic injuries and primarily affect the upper limbs [37,38]; in our study, the predominant finding is that fractures of the long bones of the upper limb constitute 54% of the cases.

Pediatric fractures differ from those in adults due to skeletal immaturity and bone physiology [8]. Fortunately, children have advantages such as remodeling capacity and the ability to avoid long-term deformities [38]. However, it’s essential to consider several prognostic factors, including the child’s age (with younger children more likely to experience significant deformities and dysmetria). In our study, we observed that patients aged 0–4 years were significantly more likely to sustain injuries at the upper arm, elbow, and upper leg compared to the lower arm (19.5%, 20.3%, and 16.1% vs. 1%, respectively). Conversely, patients aged 15–17 years were significantly more likely to sustain injuries in the upper arm compared to the lower arm (39% vs. 14.3%) (*p* < 0.001). Also the energy of the trauma, the type and severity of fractures (especially those affecting growth plates, which may impact future growth and development), skin integrity, the degree of bone exposure, the presence of vascular or nerve branch lesions, the quality of fracture reduction (if applicable), and the type of treatment (conservative or surgical).

Growth disorders are frequently observed as a consequence of premature growth plate closure or rapid partial growth, resulting in shortening or deformity of the affected bone segment. In our study, a significant finding regarding the frequency of complications was noted. Out of the total 358 cases examined, 62 (17.3%) exhibited complications. Complications occurred in 17.3% of patients, most commonly surgical (24 cases, 38.7%), orthopedic (17 cases, 27.4%), and neurological (15 cases, 24.2%).

It has been observed that younger children tend to recover better after injury compared to older children [14,16,39]. However, any disability acquired during childhood is always critical due to the potential losses, which depend on the developmental phase when the trauma occurs and have long-term implications throughout the remaining lifespan [8].

Trauma during the age of 10–14 years was the most prevalent in our study (N = 163, 45.5%) and can hinder the acquisition of expected capabilities. Additionally, trauma can have extensive repercussions on different facets of life, such as school, social activities, and the personal and professional lives of parents. These consequences may include absenteeism, alterations in educational settings, reduced participation in extracurricular activities, and disruptions to parents’ schedules and careers [40,41,42].

The findings of this study underscore the importance of implementing safety measures to protect children in their capacities as pedestrians, cyclists, or passengers in vehicles. Nonetheless, additional research is needed to identify the most effective preventive measures.

Bharat K. Soni et al. wrote: “(…) little attention has been paid to detailed modeling of the brain, which lags behind the complexity of currently published adult brain models. None of the published pediatric head models have utilized different properties to represent grey and white matter, although this distinction has been reported to influence injury prediction in the adult” [43,44]. In accordance with the literature, our study also revealed that traumatic brain injuries (52 cases, 14.5%) are not uncommon in children. Therefore, there is a critical need for future prediction models specifically addressing brain injuries.

Despite the legal requirements in Romania, which state in “ART 97, paragraph 1: Children up to 135 cm in height can be transported in vehicles equipped with safety systems for drivers and passengers, hereinafter referred to as safety systems, only if they are fixed or secured using a child seat attachment device installed in the vehicle”, we found that the majority of car passengers did not wear seatbelts or were not provided with special car seats and were instead held by their parents (n = 50, 69.5%). Additionally, Article 160, paragraph 4, recommends that, when traveling on public roads, cyclists wear approved protective helmets. In our study, we found that 69.6% (62 out of 89) of cyclists were not wearing helmets at the time of the impact [45].

Penalties should aim to encourage behavioral changes without being excessively restrictive, and there should be a robust enforcement infrastructure alongside the law itself. In addition to legislative support, organized groups play a vital role in driving legislative success [46]. Effective enforcement of helmet laws depends on several factors, including well-crafted legislation, dedicated organizational bodies for implementation, community support, accessibility to affordable helmets, and a recognized need for change [47,48]. Authorities must also impose mandatory seatbelt use and enforce the use of seatbelts. Consequently, policymakers must strike a balance when establishing enforcement policies, considering both the severity of fines or penalties and potential unintended consequences [49].

## 5. Limitations of This Study and Further Studies

Several limitations of the current study need to be acknowledged.

First, because this is retrospective research, particularly one based on a regional registry, we anticipate coding errors and missing data. Nevertheless, we used logic check tools to confirm that the database variables were coherent.

Secondly, our study is constrained by the relatively moderate number of cases among children, as our sample consists of cases throughout the entire region of Moldova, which is located in the eastern part of Romania and is the third-largest region in the country by both area and population. It also contains only hospitalized patients, predominantly the most severe cases.

Given the significant differences observed among the four age groups, especially the unique characteristics seen in ages 0–4 and 15–17, we advocate for further research specifically targeting these age groups. Expanding the study to a multicenter level could substantially enhance the value of our current data. We are considering undertaking multicenter data research in the future, despite the considerable amount of work and data correlation it entails.

## 6. Conclusions

According to our findings, age has a significant effect on the type of accident (*p* < 0.001). Patients aged 0–4 years were more frequently car passengers than pedestrians or bicyclists (28.8% vs. 9.2%/3.4%), while patients aged 15–17 years were more frequently cyclists than car passengers (28.1% vs. 8.2%) (*p* < 0.001).

Patients aged between 0 and 4 years were significantly more associated with lesions at the upper arm, elbow, and upper leg than at the lower arm (19.5%/20.3%/16.1% vs. 1%), while patients in the opposite age group of 15–17 years were significantly more associated with lesions at the upper arm than at the lower arm (39% vs. 14.3%) (*p* < 0.001).

The majority of patients underwent orthopedic treatment (47.8%) or surgical intervention (44.1%), with an average hospitalization duration of 8.5 ± 6.6 days (median = 7 days). Also, differences in hospitalization period between age categories were significant according to the Kruskal–Wallis H test (*p* = 0.043), and post-hoc Dunn–Bonferroni tests showed that only patients aged between 15 and 17 years had a significantly higher hospitalization period than patients aged between 0 and 4 years.

Complications occurred in 17.3% of patients, most commonly surgical (24 cases, 38.7%), orthopedic (17 cases, 27.4%), and neurological (15 cases, 24.2%).

## Figures and Tables

**Table 1:** Comparison and distribution of patient’s age according to gender.

Gender		Mean ± SD	Median (IQR)	Mean Rank	*p* *
Female (*p* < 0.001 **)		11.48 ± 4.08	12 (9.25–14)	185.23	0.573
Male (*p* < 0.001 **)		11.03 ± 4.45	12 (8–14)	177.85	
**Gender/Age**	**Female**		**Male**		*p* ***
	**Nr.**	**%**	**Nr.**	**%**	
0–4 years	9	11.3%	32	11.5%	0.435
5–9 years	11	13.8%	58	20.9%	
10–14 years	42	52.5%	121	43.5%	
15–17 years	18	22.5%	67	24.1%	

* Mann–Whitney U test, ** Shapiro–Wilk test, *** Fisher’s exact test.

**Table 2:** Associations between age, gender, and type personal pathological history for female patients.

Age/Personal Pathological History (Female)	Absent			Present			*p* *
	Nr. (%)			Nr. (%)			
0–4 years	8 (12.3%)			1 (6.7%)			0.416
5–9 years	7 (10.8%)			4 (26.7%)			
10–14 years	34 (52.3%)			8 (53.3%)			
15–17 years	16 (24.6%)			2 (13.3%)			
Age/Type of History (Female)	Orthopedic	Neurologic	Hematologic	Cardiologic	Allergology	Other	*p* *
	Nr. (%)	Nr. (%)	Nr. (%)	Nr. (%)	Nr. (%)	Nr. (%)	
0–4 years	0 (0%)	1 (33.3%)	0 (0%)	0 (0%)	0 (0%)	0 (0%)	0.353
5–9 years	1 (20%)	1 (33.3%)	0 (0%)	0 (0%)	2 (50%)	0 (0%)	
10–14 years	4 (80%)	0 (0%)	1 (100%)	1 (50%)	2 (50%)	0 (0%)	
15–17 years	0 (0%)	1 (33.3%)	0 (0%)	1 (50%)	0 (0%)	0 (0%)	

* Fisher’s exact test.

**Table 3:** Associations between age, gender, and type personal pathological history for male patients.

Age/Personal Pathological History (Male)	Absent			Present			*p* *
	Nr. (%)			Nr. (%)			
0–4 years	29 (12%)			3 (8.1%)			0.791
5–9 years	50 (20.7%)			8 (21.6%)			
10–14 years	106 (44%)			15 (40.5%)			
15–17 years	56 (23.2%)			11 (29.7%)			
Age/Type of history (Male)	Orthopedic	Neurologic	Hematologic	Cardiologic	Allergology	Other	*p* *
	Nr. (%)	Nr. (%)	Nr. (%)	Nr. (%)	Nr. (%)	Nr. (%)	
0–4 years	0 (0%)	1 (8.3%)	2 (28.6%)	0 (0%)	0 (0%)	0 (0%)	0.482
5–9 years	2 (28.6%)	1 (8.3%)	2 (28.6%)	0 (0%)	1 (25%)	2 (66.7%)	
10–14 years	4 (57.1%)	4 (33.3%)	2 (28.6%)	3 (75%)	1 (25%)	1 (33.3%)	
15–17 years	1 (14.3%)	6 (50%)	1 (14.3%)	1 (25%)	2 (50%)	0 (0%)	

* Fisher’s exact test.

**Table 4:** Multinomial logistic regression model used in prediction of accident types using age classes.

Accident Type/Age Group		OR (95% C.I.)	*p*
Other type	0–4 years	0.364 (0.093–1.425)	0.147
	5–9 years	0.340 (0.127–0.907)	**0.031**
	10–14 years	0.316 (0.141–0.709)	**0.005**
	15–17 years	-	-
Car passenger	0–4 years	8.5 (2.829–25.542)	**<0.001**
	5–9 years	1.943 (0.655–5.765)	0.231
	10–14 years	2.752 (1.054–7.186)	**0.039**
	15–17 years	-	-
Bicyclist	0–4 years	0.291 (0.076–1.124)	0.073
	5–9 years	0.583 (0.263–1.291)	0.183
	10–14 years	0.894 (0.473–1.689)	0.729
	15–17 years	-	-

**Table 5:** Associations between age and type of accidents.

Age/Type of Accident	Pedestrian		Other Types		Car Passenger		Bicyclist		*p* *
	Nr.	%	Nr.	%	Nr.	%	Nr.	%	
0–4 years	**14**	**9.2%**	**3**	**7%**	**21**	**28.8%**	**3**	**3.4%**	**<0.001**
5–9 years	35	22.9%	7	16.3%	12	16.4%	15	16.9%	
10–14 years	70	45.8%	13	30.2%	34	46.6%	46	51.7%	
15–17 years	**34**	**22.2%**	**20**	**46.5%**	**6**	**8.2%**	**25**	**28.1%**	

* Fisher’s exact test.

**Table 6:** Associations between gender and type of accidents.

Gender/Type of Accident	Pedestrian		Other Types		Car Passenger		Bicyclist		*p* *
	Nr.	%	Nr.	%	Nr.	%	Nr.	%	
Female	44	28.8%	6	14%	13	17.8%	17	19.1%	0.090
Male	109	71.2%	37	86%	60	82.2%	72	80.9%	

* Fisher’s exact test.

**Table 7:** Frequency of complications that occurred post-treatment in studied patients.

Complications	N	%
Without complications	296	82.7
Postoperative wound dehiscence	10	2.8
Wound infection	10	2.8
Genu valgum	8	2.2
Limping gait	8	2.2
Radial nerve palsy	7	1.9
Secondary sciatic nerve palsy	4	1.1
Acute posthemorrhagic anemia	4	1.1
Delayed union	3	0.8
Genu varum	2	0.6
Posttraumatic shortening 2.5 cm	2	0.6
Posttraumatic shortening 4.5 cm	2	0.6
Total	358	100.0

N—Number of cases, %—Percentage of the total number of cases.

## Data Availability

The data presented in this study are available on request from the corresponding author. The data are not publicly available due to specific ethical and privacy considerations.
